# Supplementary material for: Topological control of liquid-metal-dealloyed structures
Source: Nat Commun. 2022 May 25;13:2918. doi: 10.1038/s41467-022-30483-5 (PMC9133020; doi:10.1038/s41467-022-30483-5)
Supplement: Supplementary file 1 — Supplementary Information [file 41467_2022_30483_MOESM1_ESM.pdf]

Supplementary Information

Topological Control of Liquid-Metal-Dealloyed Structures

L. Lai et al.

1 Supplementary Methods: Phase field method

We use phase-field method to model the ternary alloy dealloying process. For a ternary alloy system, we use c_1, c_2 and c_3 to represent the concentration of Cu, Ti and Ta, respectively, with the constraint $c_1 + c_2 + c_3 = 1$. We introduce a phase field ϕ to distinguish between the liquid ($\phi = 0$) and solid ($\phi = 1$) phases. The total free-energy is defined as [1],

$$F = \int_V \mathrm{d}V \left[\frac{\sigma_\phi}{2} |\nabla \phi|^2 + f(\phi) + \sum_i \frac{\sigma_i}{2} |\nabla c_i|^2 + f_c(\phi, c_1, c_2, c_3) \right]. \tag{1}$$

The first two terms in the integrand of F include a gradient energy term and a double-obstacle potential with two minima at 0 and 1 defined by

$$\begin{aligned} f(\phi) &= +\infty & &\text{for } \phi < 0 \\ f(\phi) &= \lambda_\phi \phi(1-\phi) & &\text{for } 0 \leq \phi \leq 1 \\ f(\phi) &= +\infty & &\text{for } \phi > 1 \end{aligned} \tag{2}$$

where the parameters λ_ϕ and σ_ϕ are chosen to obtain a solid-liquid interface width $w_i = 2$ nm and an excess free-energy $\gamma = 0.2$ J/m^2. The third term is the sum of gradient energy terms that represent the excess free-energy associated with the formation of compositional domain boundaries. The fourth term is the chemical contribution to the bulk free-energy density that determines the thermodynamic properties of the ternary alloy. It is assumed to have the form

$$f_c(\phi, c_i) = \sum_{i=1}^{3} \left(\phi c_i L_i \left(\frac{T - T_i}{T_i} \right) + \frac{k_B T}{V_a} c_i \log(c_i) \right) + \sum_{i<j}^{i,j\leq 3} c_i c_j (\phi L_{ij}^s + (1-\phi) L_{ij}^l) \tag{3}$$

The first part couples the concentration fields to the phase field through the temperature T, the melting point of the compound T_i and the latent heat of pure elemental systems L_i. The second part is the entropy term of each element. We assume that the atomic volume V_a is the same for all the elements and does not change between the solid and liquid phase. The last part is the mixing enthalpy which is expressed in a Redlich-Kister (RK) power series [2] for both the solid and liquid

$$L_{ij} = \sum_{\nu=0}^{k} (c_i - c_j)^\nu \cdot {}^\nu L_{ij}. \tag{4}$$

For the Cu-Ti-Ta ternary system, we only use the lowest order $\nu = 0$ where $\Omega_{ij} = {}^0L_{ij}^s = {}^0L_{ij}^l$.

The evolution equations of the order parameters can be derived from the variations in the total free energy. The concentrations c_1 and c_2 are conserved order parameters, which follow Cahn-Hilliard-like equations of the form [3]

$$\dot{c_i} = \nabla \cdot M_{ij} \nabla \mu_j \tag{5}$$

where $\mu_j = \delta \mathcal{F} / \delta c_j$ denotes the chemical potential of element c_j and M_{ij} are the elements of the mobility matrix that is symmetric due to Onsager reciprocal relations. These elements are expressed as

$$M_{ij} = M_0(\phi) c_i (\delta_{ij} - c_j), \tag{6}$$

where $M_0(\phi) = (1 - \phi(x)) M_l + \phi(x) M_s$ is a linear function of ϕ which is chosen such that the mobility in the solid is M_s and in the liquid is M_i. The values of M_i and M_s depend on the

diffusion coefficients in the liquid phase $M_l = D_l V_a/kT$ and the solid phase $M_s = D_s V_a/kT$ [4], where V_a is the atomic volume and D_l (D_s) is the liquid (solid) diffusivity. The evolution equation for the phase field has the standard form ϕ [5] :

$$\dot{\phi} = -L_\phi \frac{\delta \mathcal{F}}{\delta \phi} \tag{7}$$

We assume that the kinetics of the solid-liquid interface is fast on a diffusive time scale so that the interface is in local thermodynamic equilibrium. Accordingly, we choose the normalized coefficient $L_\phi w_i^2/M_l = 10$ in the simulations large enough to achieve this limit while keeping the simulations computationally tractable. The other parameters used in our simulations are shown in the following (in the order of Cu, Ti, Ta):

$T_{\rm sim} = 1775\,\rm K$, $V_a = 0.01\,\rm nm^3$, $\sigma_\phi = 3.18\,\rm eVnm^{-1}$, $\lambda_\phi = 1.59\,\rm eVnm^{-3}$

$L_1 = 11.5\,\rm eVnm^{-3}$, $L_2 = 11.8\,\rm eVnm^{-3}$, $L_3 = 17.6\,\rm eVnm^{-3}$

$T_1 = 1358\,\rm K$, $T_2 = 1941\,\rm K$, $T_3 = 3290\,\rm K$

$\sigma_1 = \sigma_2 = \sigma_3 = 9.0\,\rm eVnm^{-1}$

$D_{l1} = D_{l2} = D_{l3} = 7 \times 10^9\,\rm nm^2s^{-1}$, $L_\phi = 1.14 \times 10^9\,\rm nm^3eV^{-1}s^{-1}$

$\Omega_{12} = \Omega_{23} = 0$, $\Omega_{13} = 90\ \rm eVnm^{-3}$

The temperature of dealloying in experiments is $T_{\rm exp} = 1513$ K. As in our previous study [1], we use a higher temperature $T_{\rm sim} = 1775$ K to quantitatively reproduce essential features of the experimental phase diagrams at 1513 K. Specifically, the solubility range of Ti in the Cu melt in experiments at $T_{\rm exp}$ is the same as in simulations at $T_{\rm sim}$. We assume that convection in the liquid bath is negligible and that consequently the diffusive boundary layer outside the dealloyed structure, which increases in width with time, can be arbitrarily large [6]. To make the computations efficient, we use 2D or 3D grids that extend a finite distance into the melt outside the dealloyed layer where the concentration fields become spatially uniform along a line (in 2D) or plane (in 3D) parallel to the initial planar solid-liquid interface at the start of dealloying. Only 1D diffusion equations need to be solved in the melt for larger distances and the concentration fields on the 1D and 2D/3D grids are matched at the interface between 1D and 2D/3D grids. The 1D liquid layer is chosen large enough for the concentrations to remain constant far from the dealloyed layer. 2D and 3D simulations are carried out with $dx/w_i = 0.25$ and dtD_l/w_i^2 equal to 0.00015 and 0.00008 in 2D and 3D, respectively.

2 Supplementary Note: Phase diagram

2.1 Phase diagram of Cu-Ti-Ta ternary system

For a ternary system, the conditions for two-phase solid-liquid coexistence consist of equalities of chemical potentials of two species (since $c_1 + c_2 + c_3 = 1$) and equality of grand potential

$$\mu_1^s(c_i^s) = \mu_1^l(c_i^l), \tag{8}$$

$$\mu_2^s(c_i^s) = \mu_2^l(c_i^l), \tag{9}$$

$$f^s(c_i^s) - c_1^s\mu_1^s(c_i^s) - c_2^s\mu_2^s(c_i^s) = f^l(c_i^l) - c_1^l\mu_1^l(c_i^l) - c_2^l\mu_2^l(c_i^l) \tag{10}$$

where the solid $f^s(c_i^s) = f(1, c_i^s)$ and liquid $f^l(c_i^l) = f(0, c_i^l)$ free energies are obtained from Equation 3, and $\mu_i^{s,l}(c_i^{s,l}) = \delta f^{s,l}(c_i)/\delta c_i|_{c_i=c_i^{s,l}}$ are the chemical potentials. From the given thermodynamic parameters, we can solve the equilibrium equations with only one independent variable left. The result is shown in Supplementary Figure 1a. Due to the large mixing enthalpy between Cu and Ta, the Ta solubility in the Cu melt is very small. However, there is no

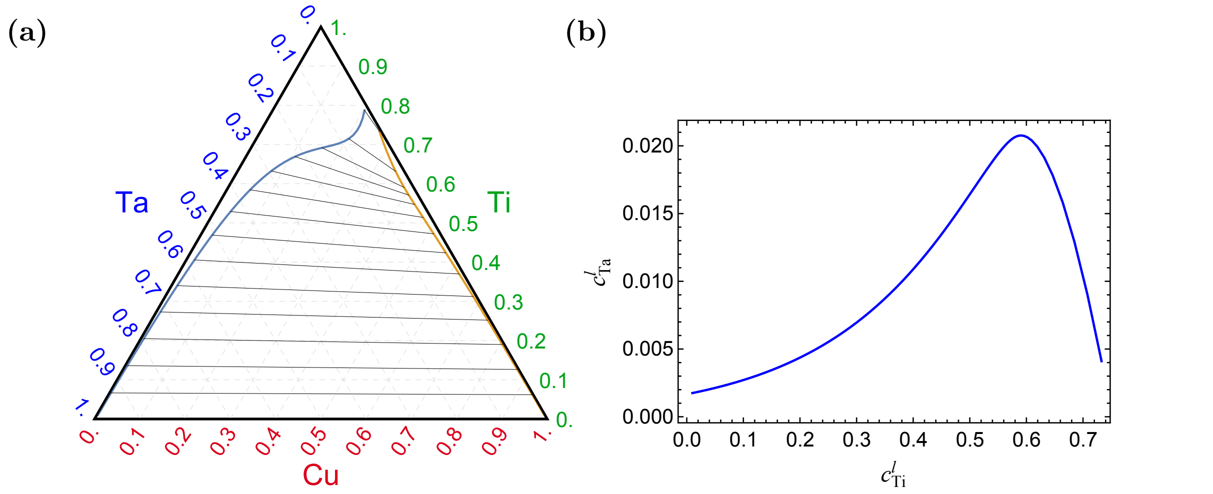

Supplementary Figure 1. (a) Cu-Ti-Ta ternary phase diagram showing the regions of two-phase coexistence between the solidus (blue line) and the liquidus (organge line). (b) Different representation of the liquidus line showing the Ta solubility in the Cu-Ti melt versus the concentration of Ti in the liquid.

mixing enthalpy between Ta and Ti, so the Ta solubility in the Cu-Ti melt increases with Ti concentration (Supplementary Figure 1b).

2.2 Modeling the interaction between Cu-Ag and Ti

From experimental phase diagrams, the solubility of Ti in Ag at 1513 K is around 14.6%. To reproduce this solubility, we choose the mixing enthalpy of the Ag-Cu-Ti system as $\Omega_{\mathrm{AgCu}} = \Omega_{\mathrm{CuTi}} = 0$ and $\Omega^s_{\mathrm{AgTi}} = 24\,\mathrm{eV/nm}^3$, $\Omega^l_{\mathrm{AgTi}} = 33\,\mathrm{eV/nm}^3$. The interaction between Ag, Ti and Cu are shown in a ternary phase diagram in Supplementary Figure 2(a). If we initially dissolve some Ag in the pure Cu melt, we can expect that the ratio of the compositions of Ag and Cu ($c_{\mathrm{Ag}}/c_{\mathrm{Cu}}$) is constant when Ti is dissolved during dealloying. Therefore, we can calculate the solubility of Ti in an arbitrary $\mathrm{Cu}_{1-x}\mathrm{Ag}_x$ melt by calculating the concentration of Ti at the point of intersection of the fixed ratio line and liquidus. As shown in Supplementary Figure 2(a), the intersection of a line with a ratio Ag:Cu=3:7 with the liquidus gives the solubility of Ti ($c_l = 0.5732$) in a $\mathrm{Cu}_{70}\mathrm{Ag}_{30}$ melt.

In phase-field modeling, we introduce a pseudo-element $\mathrm{Cu}'(x)$=$\mathrm{Cu}_{1-x}\mathrm{Ag}_x$ to replace the $\mathrm{Cu}_{1-x}\mathrm{Ag}_x$ melt. We can vary the mixing enthalpy of the effective Ti-Cu$'$ system to reach the solubility of Ti in the Cu-Ag melt. The mixing enthalpy of Ti-($\mathrm{Cu}_{70}\mathrm{Ag}_{30}$) is ${}^0L^s_{\mathrm{Cu'Ti}} = 18.6\ \mathrm{eV/nm}^3$, ${}^0L^l_{\mathrm{Cu'Ti}} = 20\ \mathrm{eV/nm}^3$, ${}^1L^s_{\mathrm{Cu'Ti}} = -7\ \mathrm{eV/nm}^3$, ${}^1L^l_{\mathrm{Cu'Ti}} = -7.7\ \mathrm{eV/nm}^3$. The corresponding binary phase diagram is shown in Supplementary Figure 2(b).

2.3 Modeling the interaction between Cu-Ag and Ta

Ta is considered immiscible with Cu at the experimental dealloying temperature. However, the solubility of Ta is non-zero in molten Cu. When the mixing enthalpy $\Omega_{13} = 90\ \mathrm{eV/nm}^3$, the solubility of Ta is 1.7×10^{-3}. There is no report about the exact solubility of Ta in Ag, but the solubility in Ag is much smaller than in Cu. The mixing enthalpies for the Ag-Cu-Ti system can be estimated to be $\Omega_{\mathrm{AgCu}} = 0$, $\Omega^s_{\mathrm{AgTa}} = 90\ \mathrm{eV/nm}^3$, $\Omega^l_{\mathrm{AgTa}} = 160\ \mathrm{eV/nm}^3$, and $\Omega_{\mathrm{CuTa}} = 90\ \mathrm{eV/nm}^3$, which yields a solubility of Ta in molten Ag of 1.69×10^{-5}. The

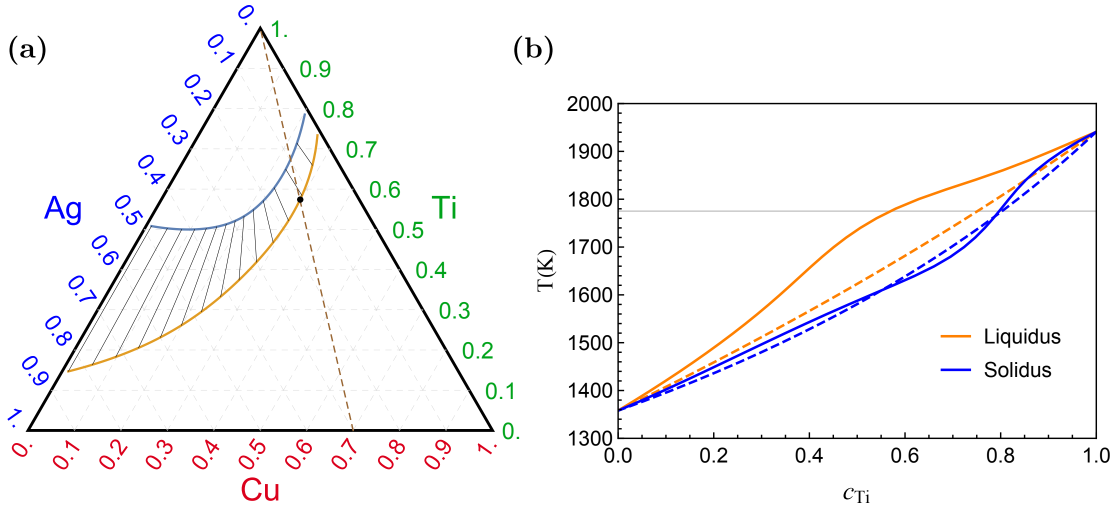

Supplementary Figure 2. (a) Ti-Cu-Ag ternary phase diagram with solidus (blue line) and liquidus (organge line). The brown dashed line corresponds to a fixed ratio (Ag:Cu=3:7). (b) Ti-Cu phase diagram. The dashed line is the original Ti-Cu phase diagram. The solid line is the adapted Ti-Cu′ phase diagram with new mixing enthalpy to simulate the Ti-($Cu_{70}Ag_{30}$) phase diagram.

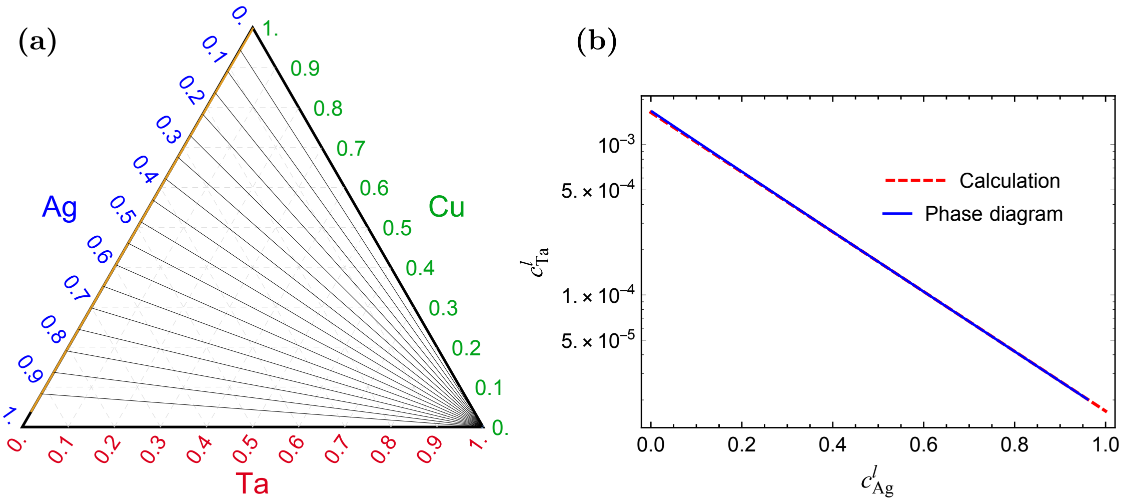

Supplementary Figure 3. (a) Ta-Cu-Ag ternary phase diagram. Since the solubilities of Ta in Ag and Cu are small, the solidus line is near the pure Ta bottom right corner of the Gibbs triangle and the liquidus (organge line) is near the left edge. (b) Plot of Ta solubility versus Ag concentration in the melt. The numerically calculated line from the phase diagram is shown (blue line) is in very good quantitative agreement with the analytical prediction of Equation 11 (red dashed line).

corresponding ternary phase diagram is shown in Supplementary Figure 3(a).

In the dilute limit that $c^l_{Ta} \to 0$, $c^s_{Ta} \to 1$ and $c^s_{Ag} \to 0$, we can calculate analytically the liquidus from the two-phase equilibrium conditions (Equations 8-10), which yields the prediction shown in Supplementary Figure 3(b)

$$c^l_{\mathrm{Ta}} = \mathrm{e}^{\frac{V_a}{k_B T}\left(L_{\mathrm{Ta}}\frac{T-T_{\mathrm{Ta}}}{T_{\mathrm{Ta}}} - \Omega^l_{\mathrm{TaAg}} c^l_{\mathrm{Ag}} - \Omega^l_{\mathrm{TaCu}} c^l_{\mathrm{Cu}}\right)}. \tag{11}$$

This solubility only depends on the ratio of the composition of Cu-Ag. If we define as before a pseudo-element $\mathrm{Cu}'(x)$ to replace the Ag-Cu melt, the Ta solubility in the dilute limit of the equivalent Ta-$\mathrm{Cu}'(x)$ binary phase diagram becomes

$$c^l_{\mathrm{Ta}} = \mathrm{e}^{\frac{V_a}{k_B T}\left(L_{\mathrm{Ta}}\frac{T-T_{\mathrm{Ta}}}{T_{\mathrm{Ta}}} - \Omega^l_{\mathrm{TaCu}'(x)}\right)}, \tag{12}$$

where $\Omega^l_{\mathrm{TaCu}'(x)} = \Omega^l_{\mathrm{TaAg}} x + \Omega^l_{\mathrm{TaCu}}(1-x)$ and $x = c^l_{\mathrm{Ag}}$. For the $\mathrm{Cu}_{70}\mathrm{Ag}_{30}$ melt, the effective mixing enthalpy is $\Omega^l_{\mathrm{TaCu}'(0.3)} = 111\ \mathrm{eV/nm}^3$ and the solubility of Ta is 4.2×10^{-4}.

2.4 Phase diagram of (CuAg)-Ti-Ta ternary system

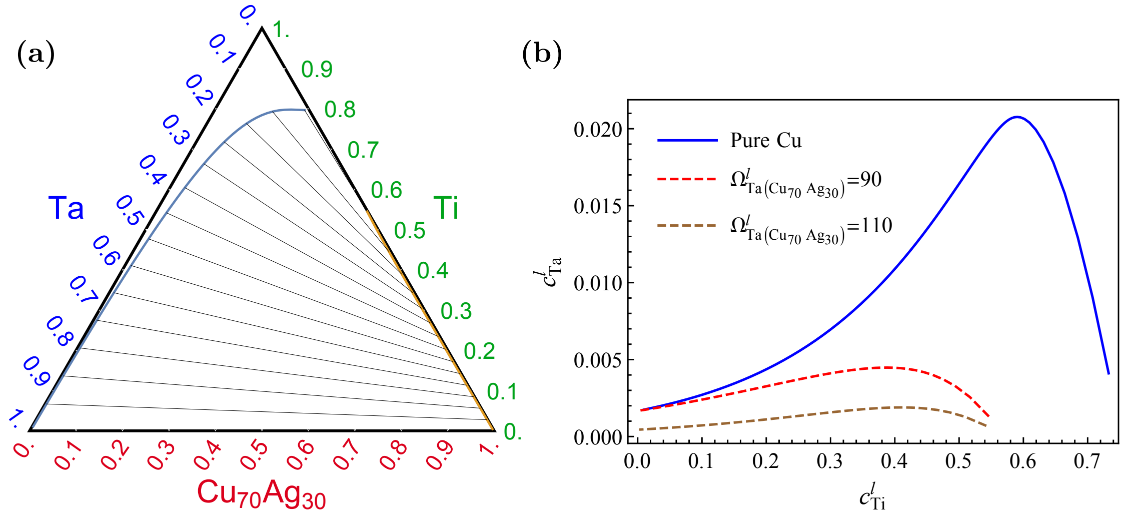

Supplementary Figure 4. (a) Effective $(\mathrm{Cu}_{70}\mathrm{Ag}_{30})$-Ti-Ta ternary phase diagram showing the regions of two-phase coexistence between the solidus (blue line) and the liquidus (organge line). (b) Plots of Ta solubility versus Ti concentration x in $\mathrm{Cu}_{1-x}\mathrm{Ti}_x$ (blue line) and $(\mathrm{Cu}_{70}\mathrm{Ag}_{30})_{1-x}\mathrm{Ti}_x$ (dashed lines) melts.

From the results of the previous two sections, we can use a pseudo-element Cu' to replace Ag and Cu in the quaternary system. Therefore we can plot a ternary phase diagram of Cu-Ag-Ti-Ta quaternary system (Supplementary Figure 4a). While we only consider the effect of Ti solubility (from Supplementary Note 2.2), the phase diagram in Supplementary Figure 4a shows that the equilibrium concentration of Ti in the liquid is lower for the same concentration of Ta in the solid. We can further expect the Ta solubility is decreased as the Ti content in the Cu melt is reduced (Supplementary Figure 4b). Those effects are adequate to promote the formation of topologically connected structures. All the simulations of the Cu-Ag melt shown in this study model the interaction between Cu-Ag and Ti (Supplementary Note 2.2) with $\Omega_{\mathrm{TaCu}'} = 90\ \mathrm{eV/nm}^3$. The effect of varying the mixing enthalpy of Cu'-Ta ($\Omega_{\mathrm{TaCu}'}$) only changes the solubility of Ta (Supplementary Figure 4b), which further reduces the coarsening and leak rate.

3 Supplementary Note: Concentration profiles

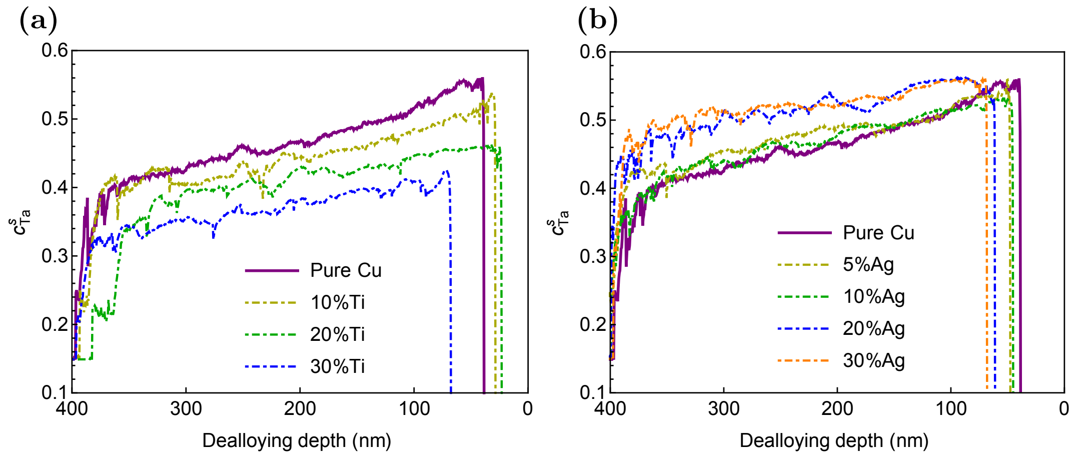

Supplementary Figure 5. Ta concentration profiles of the solid branches with different melt compositions by adding Ti (a) and adding Ag (b).

In this section, we first provide the supplemental concentration profiles of Ta in the solid branches from the phase-field simulations to support the comparison between the phase-field simulations and experiments. In ECD, the composition of the residual miscible element (e.g., Ag of the Au-Ag base alloy) in the ligaments is controlled by the dealloying kinetics [7]. In contrast, the retention of Ti in the ligaments in LMD is determined by the local chemical equilibrium with the melt composition. As shown in the previous section, the equilibrium condition for a two-phase ternary system is not fixed (Supplementary Figure 1a). While the melt composition decreases from the dealloying front to the edge (Figure 3b in the main text), the equilibrium composition of Ta in the ligaments also increases (Supplementary Figure 5). In Supplementary Figure 5a, we also find that the overall concentration of Ta in the whole ligament is lower for the higher melt composition of Ti, which indicates the more retention of Ti in the higher melt composition of Ti, thereby preserving the bicontinuous structures with larger volume fraction. For the quaternary system with Ag addition, the phase equilibrium is too complicated. For the phase-field simulations with our quasi-quaternary system, the solid Ta concentration remains roughly the same for different Ag composition in the melt while the Ti concentration in the melt decreases for the larger Ag composition (Figure 3b in the main text).

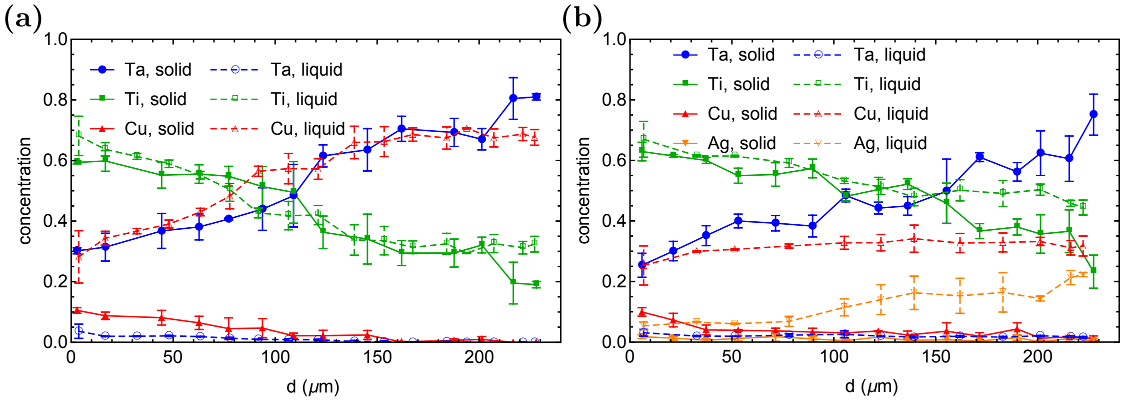

Supplementary Figure 6. Concentration profiles of dealloying in the pure Cu melt (a) and $Cu_{70}Ag_{30}$ melt (b) from experiments. $d = 0$ is the dealloying front. Error bars represent the standard deviation.

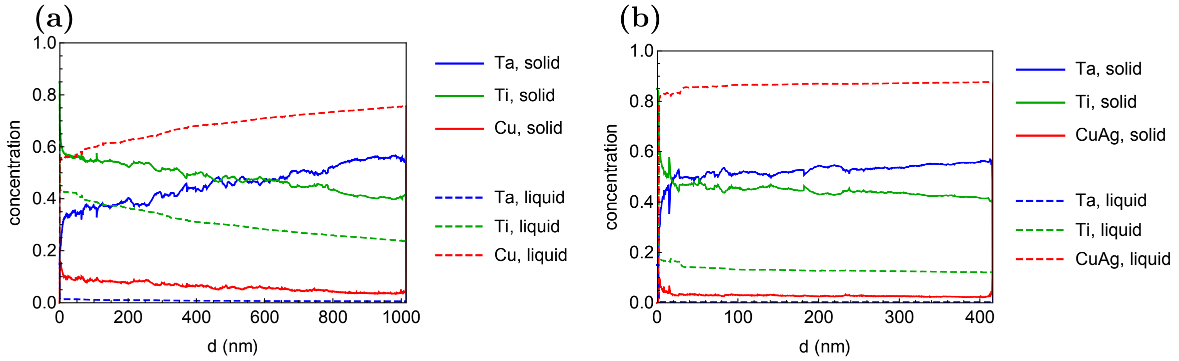

Supplementary Figure 7. Concentration profiles of dealloying in the pure Cu melt (a) and $Cu_{70}Ag_{30}$ melt (b) from phase-field simulations. $d = 0$ is the dealloying front.

To support the main text, we also provide the comprehensive concentration profiles from the dealloying in the pure Cu melt and $Cu_{70}Ag_{30}$ melt by both experiments (Supplementary Figure 6) and phase-field simulations (Supplementary Figure 7). We notice that the concentration of Cu in the solid is not zero, but varies from 10% at the dealloying front to 0% at the edge. As we discussed in the main text, the increasing concentration of Ti in the liquid also increases the solubility of Ta in the liquid due to the high miscibility of Ta and Ti. Sharing the same mechanism, Ti in the solid ligaments also increases the solubility of Cu in the ligaments, resulting in a relatively high concentration of Cu in the ligaments.

4 Supplementary Note: Dealloying kinetics

The ternary dealloying kinetics in the 2D or 3D is difficult to predict by the analytical method. However, we can estimate the dealloying kinetics based on the 1D dissolution analysis of the binary system [6]. From the 1D dissolution model, the concentration profile in the liquid is,

$$c(x,t) = c^l_{Ti} + \left(c^s_{Ti} - c^l_{Ti}\right)\sqrt{p\pi}e^p\left(\mathrm{erf}\left(-\frac{x}{\sqrt{4D_l t}}\right) - \mathrm{erf}(\sqrt{p})\right), \tag{13}$$

where D_l is the liquid diffusivity and the constant p is the Peclet number. In the infinity boundary condition ($c_\infty = c^B_{Ti}$), the Peclet number satisfies the constraint,

$$\Omega = \sqrt{\pi p}e^p(1 + \mathrm{erf}(\sqrt{p})), \tag{14}$$

where $\Omega = \frac{c^l_{Ti} - c^B_{Ti}}{c^s_{Ti} - C^l_{Ti}}$. The solid-liquid interface position is $x_i(t) = -\sqrt{4D_l pt}$.

In the 2D and 3D dealloying process, we assume the dissolution of Ta in the melt and Cu in the solid ligaments is negligible. Therefore the dealloying recovers the binary dissolution of Ti in the Cu melt. As shown in Supplementary Figure 8, the base alloy consists of Ta with composition c^0_{Ta} and Ti with composition $1 - c^0_{Ta}$, the solid Ta-rich ligaments consist of Ta with composition of c^s_{Ta} and Ti with composition of $1 - c^s_{Ta}$, and the melt consists of Ti with composition of c^l_{Ti} and Cu with composition of $1 - c^l_{Ti}$. L_l is the width of the liquid channels, and L_s is the width of the solid Ta-rich ligaments. At the dealloying front, the conservation of Ta requires that,

$$c^0_{Ta}(L_l + L_s) = c^s_{Ta}L_s. \tag{15}$$

We neglect the coarsening and any change of composition of the Ta-rich ligaments that maintain a composition c^s_{Ta} because the diffusion is frozen in the solid. On the other hand, the diffusion

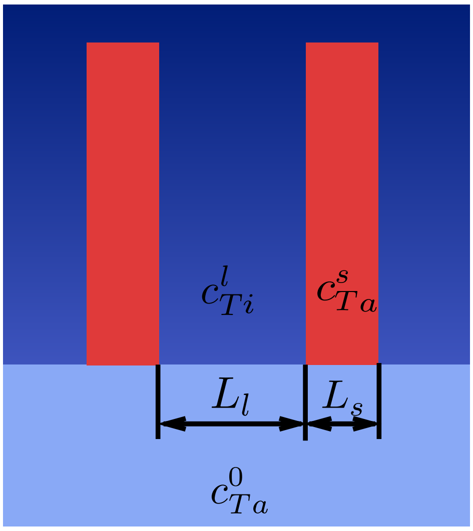

Supplementary Figure 8. A schematic diagram of the 2D dealloying. The light blue, dark blue, and red area represents the base alloy, liquid melt, and solid branches, respectively.

in the liquid channels allows the evacuation of Ti away from the dealloying front. Thus, the concentration of Ti at the interface follows the relation

$$(1 - c^0_{Ta})(L_s + L_l)v_i = v_i\left[(1 - c^s_{Ta})L_s + c^l_{Ti}L_l\right] + L_l D_l \frac{\partial c(x)}{\partial x}\bigg|_{\text{dealloying front}}, \tag{16}$$

where $c(x)$ is the concentration profile of Ti in the liquid. These two conservations yield the boundary condition,

$$v_i = \frac{D_l}{1 - c^l_{Ti}} \frac{\partial c(x)}{\partial x}\bigg|_{\text{dealloying front}} \tag{17}$$

This boundary condition is similar to the binary dissolution boundary condition Eq. A2 in the Ref.[6]. Following the same steps, we obtain the relation of Peclet number,

$$\Omega' = \sqrt{\pi p}e^p(1 + \mathrm{erf}(\sqrt{p})), \tag{18}$$

where $\Omega' = \frac{c^l_{Ti} - c^B_{Ti}}{1 - c^l_{Ti}}$. The corresponding dealloying depth is $x_i(t) = -\sqrt{4pD_l t}$.

5 Supplementary Note: Coarsening of ligaments

Supplementary Figure 9 are the examples of the cross-section of the dealloyed structure for pure Cu and $Cu_{70}Ag_{30}$ melts. The experimental data of the ligament size and spacing is summarized from several measurements. Let us consider the dealloyed structure at the distance d from the dealloying front (Supplementary Figure 10a). The dealloyed structure starts coarsening at the time $t_f(d) = (x_i - d)^2/(4pD_l)$, when the dealloying front reaches the position $(x_i - d)$. The coarsening ends at the time $t_e = x_i^2/(4pD_l)$, when the dealloying front stops at x_i. The coarsening time for the structure at distance d is $t_c(d) = t_e - t_f(d)$. According to the standard coarsening law, the evolution of the characteristic length λ follows the equation:

$$\lambda(d)^n = kt_c(d) + \lambda_{00}(d)^n, \tag{19}$$

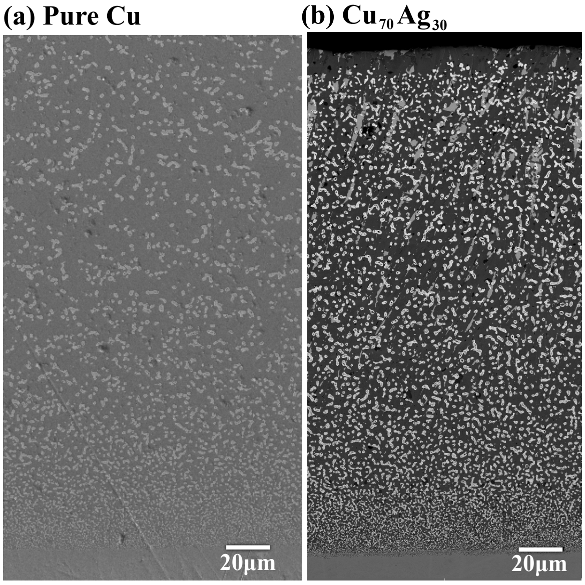

Supplementary Figure 9. The cross-section of the dealloyed structures from SEM for $Ta_{15}Ti_{85}$ base alloy dealloyed in the pure Cu melt (a) and $Cu_{70}Ag_{30}$ melt (b).

where $n = 3$ for liquid state bulk diffusion, and $n = 4$ for surface diffusion. From the previous dealloying research[1], we have the relation $\lambda_{00}(d)^2 = C_1(x_i - d)$. Therefore, Eq. 19 becomes

$$\lambda(d) = \left[\frac{k}{4pD_l}\left(2x_i d - d^2\right) + C_1^{\frac{n}{2}}\left(x_i - d\right)^{\frac{n}{2}}\right]^{\frac{1}{n}}. \tag{20}$$

The dealloying time is 10 s, and the averaged dealloying depth for the experiments is around $x_i = 240$ μm, hence the parameter $C_1 = 0.00174$ μm. In this equation, remaining unknowns are coarsening coefficient k and power n. We can fit the experimental results with this equation to obtain the parameters. However, a more straightforward way to determine the power n is Eq. 19, where $\lambda^n - \lambda_{00}^n$ has a linear dependence with t_c. Since the ligament width λ_w and ligament spacing λ_s are not equivalent during coarsening in LMD, we show separately the parametric plots of $\lambda_w^n(d) - \lambda_{w00}^n(d)$ (Supplementary Figure 10b) and $\lambda_s^n(d) - \lambda_{s00}^n(d)$ (Supplementary Figure 10b) versus $t_c(d)$ by varying d from 0 to x_i, which is equivalent to $t_c(d)$ varying from 0 s to 10 s. The result shows that both λ_w and λ_s are not linear in time for $n = 3$ or $n = 4$, which indicates that the coarsening during LMD is not bulk-diffusion or interface-diffusion capillary-driven controlled.

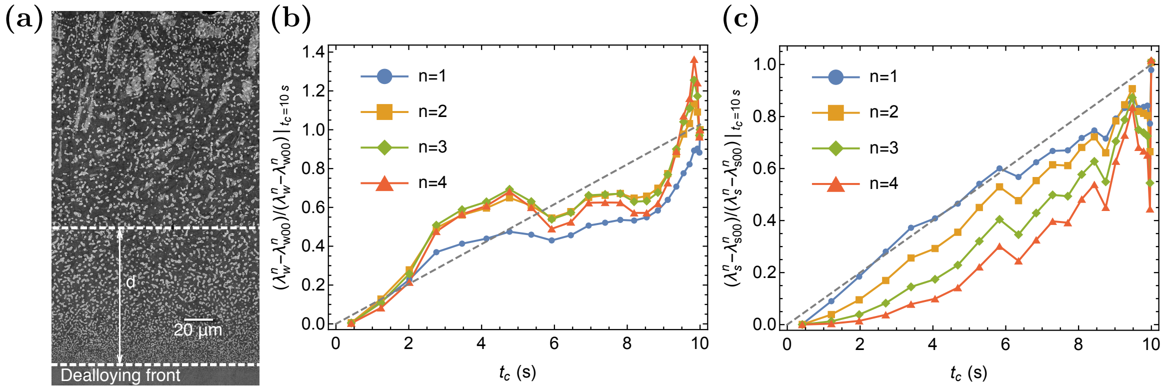

Supplementary Figure 10. (a) TEM picture from the dealloying experiment of $Ta_{15}Cu_{85}$ base alloy dealloyed in the $Cu_{70}Ag_{30}$ melt. The light area is solid, and the dark area is liquid. (b) The distribution of ligament width from the $Ta_{15}Cu_{85}$ base alloy dealloyed in the $Cu_{70}Ag_{30}$ melt. (c) The distribution of ligament spacing from the $Ta_{15}Cu_{85}$ base alloy dealloyed in the pure Cu melt. The y axis is normalized to show a fair comparison for different power laws. The dashed line in (b) and (c) is a guide to the linear dependence.

6 Supplementary References

[1] Pierre-Antoine Geslin, Ian McCue, Bernard Gaskey, Jonah Erlebacher, and Alain Karma. Topology-generating interfacial pattern formation during liquid metal dealloying. *Nature communications*, 6:8887, 2015.

[2] Hans Lukas, Suzana G Fries, and Bo Sundman. *Computational thermodynamics: the Calphad method*. Cambridge university press, 2007.

[3] John W Cahn. On spinodal decomposition. *Acta metallurgica*, 9(9):795–801, 1961.

[4] Jan-Olof Andersson and John Ågren. Models for numerical treatment of multicomponent diffusion in simple phases. *Journal of applied physics*, 72(4):1350–1355, 1992.

[5] JW Cahn and SM Allen. A microscopic theory for domain wall motion and its experimental verification in fe-al alloy domain growth kinetics. *Le Journal de Physique Colloques*, 38(C7):C7–51, 1977.

[6] Ian McCue, Bernard Gaskey, Pierre-Antoine Geslin, Alain Karma, and Jonah Erlebacher. Kinetics and morphological evolution of liquid metal dealloying. *Acta Materialia*, 115:10–23, 2016.

[7] Zhen Qi and Jörg Weissmüller. Hierarchical nested-network nanostructure by dealloying. *Acs Nano*, 7(7):5948–5954, 2013.
