# Supplementary material for: Topological control of liquid-metal-dealloyed structures
Source: Nat Commun. 2022 May 25;13:2918. doi: 10.1038/s41467-022-30483-5 (PMC9133020; doi:10.1038/s41467-022-30483-5)
Supplement: Supplementary file 3 — Description of Additional Supplementary Files [file 41467_2022_30483_MOESM3_ESM.docx]

**Description of Additional Supplementary Files**

**File Name: Supplementary Movie 1
Description:** 3D phase-field simulations of the alloy Ti85Ta15 dealloyed in the Cu70Ag30 melt.

**File Name: Supplementary Movie 2
Description:** 2D phase-field simulations of the alloy Ti85Ta15 dealloyed in the Cu70Ag30 melt.

**File Name: Supplementary Movie 3
Description:** 2D phase-field simulations of the alloy Ti85Ta15 dealloyed in the Cu70Ti30 melt.

**File Name: Supplementary Movie 4
Description:** 2D phase-field simulations of the alloy Ti85Ta15 dealloyed in the pure Cu melt.

**File Name: Supplementary Data 1
Description:** Data of experimental results.
