# Supplementary figures and images for: Topological control of liquid-metal-dealloyed structures
Source: Nat Commun. 2022 May 25;13:2918. doi: 10.1038/s41467-022-30483-5 (PMC9133020; doi:10.1038/s41467-022-30483-5)

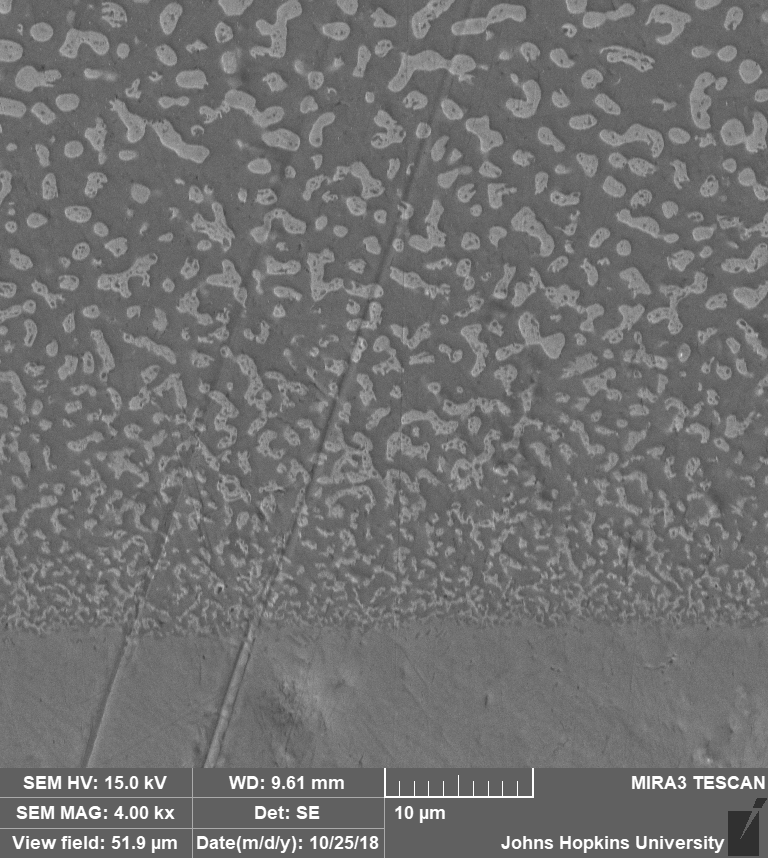

Supplement: Supplementary file 8 — Supplementary Data 1 [file 41467_2022_30483_MOESM8_ESM.zip › 20181025_Ta15Ti85-3_Cu_10s_1240_4kx_interface.tif]

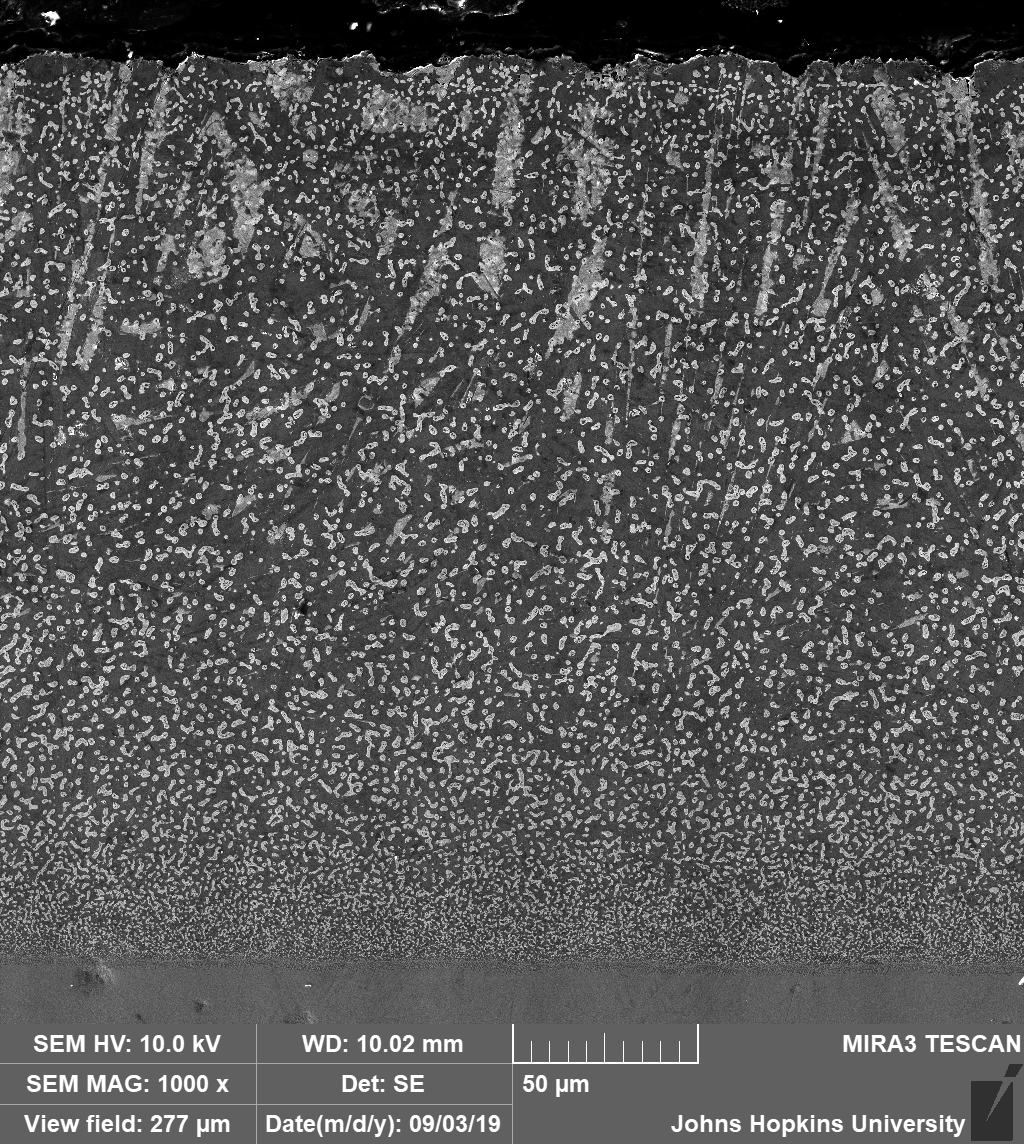

Supplement: Supplementary file 8 — Supplementary Data 1 [file 41467_2022_30483_MOESM8_ESM.zip › 20190903_Ta15Ti85-Cu70Ag30_10s_1240C_1000x.tif]

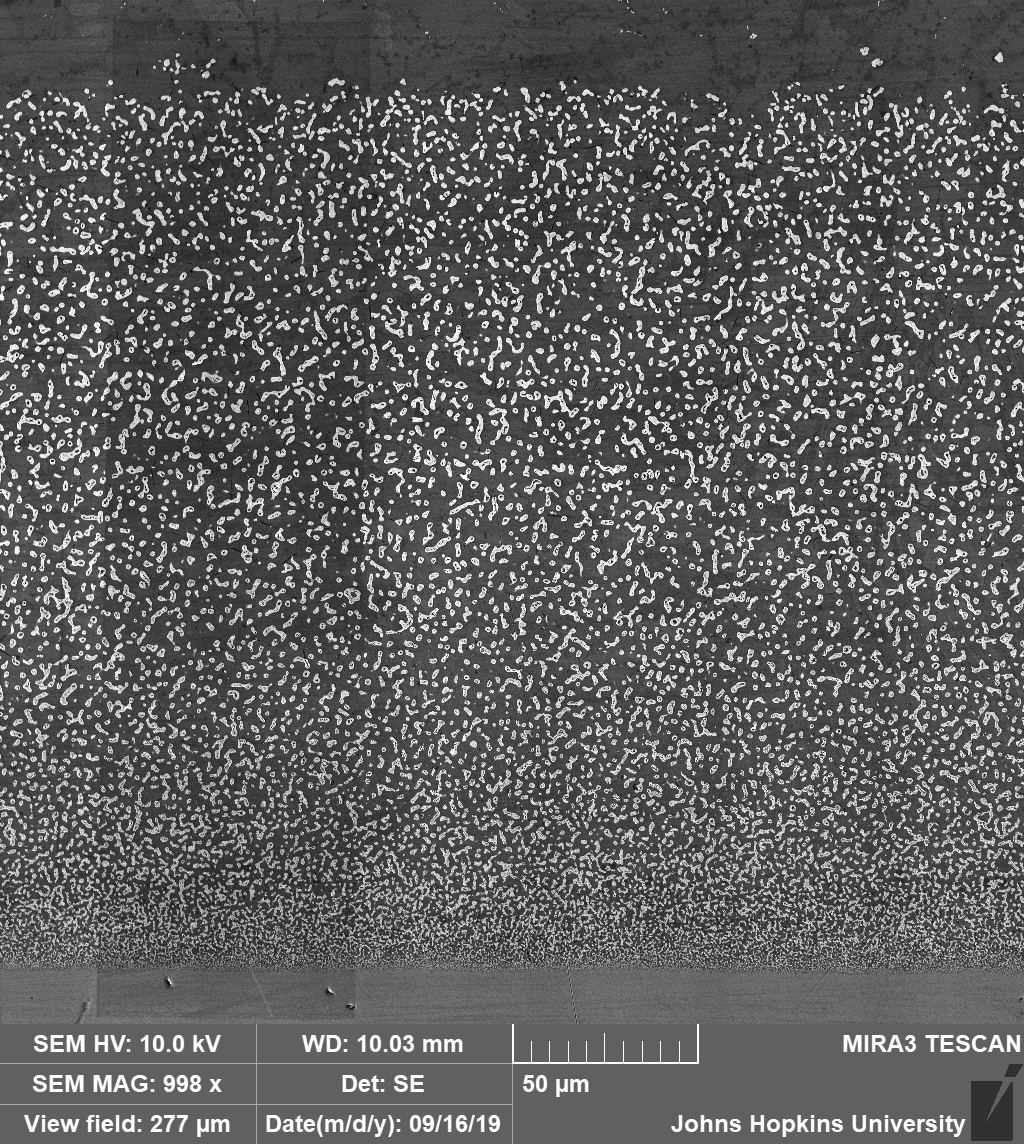

Supplement: Supplementary file 8 — Supplementary Data 1 [file 41467_2022_30483_MOESM8_ESM.zip › 20190916_Ta15Ti85-Cu_10s_1240C_998x.tif]

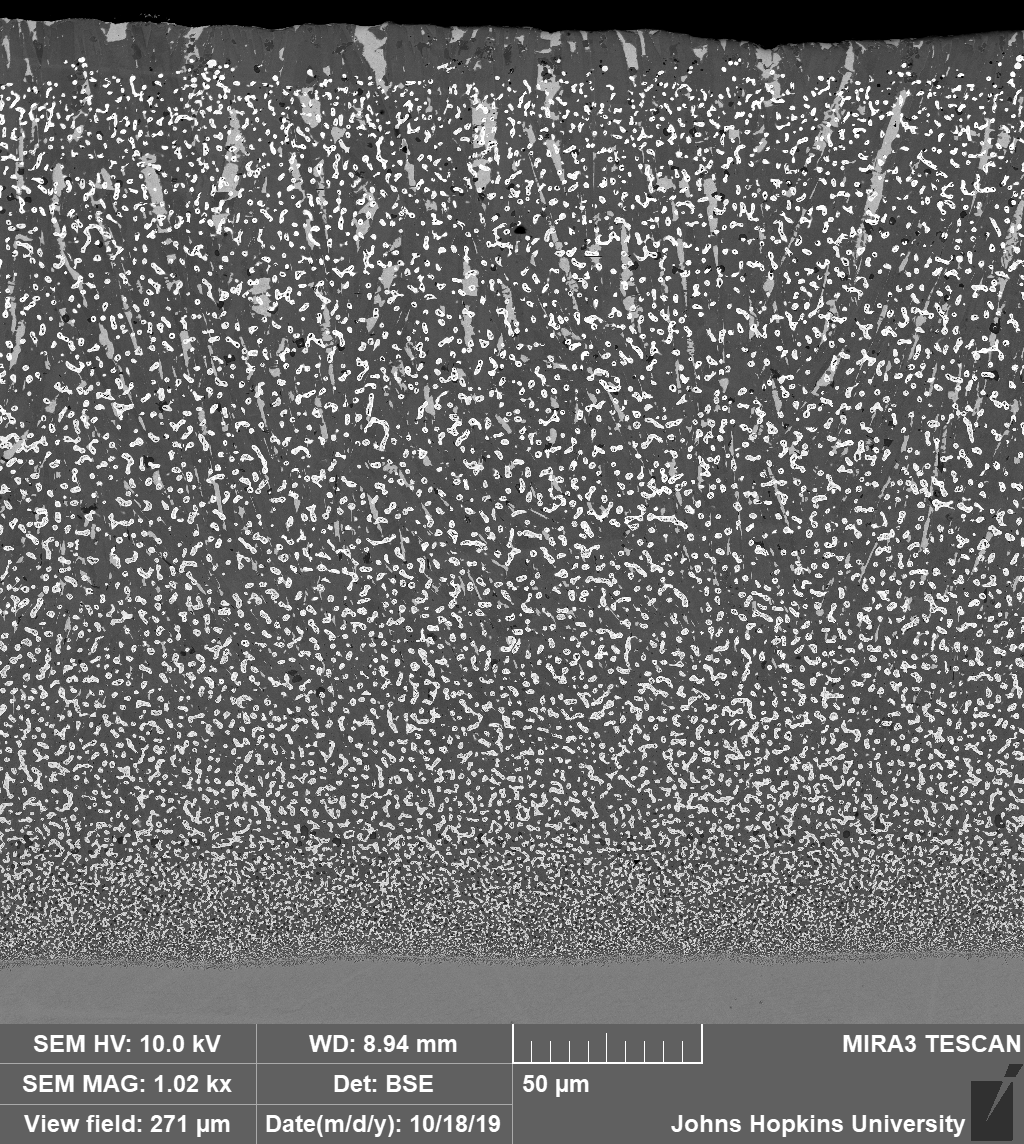

Supplement: Supplementary file 8 — Supplementary Data 1 [file 41467_2022_30483_MOESM8_ESM.zip › 20191018_Ta15Ti85-Cu70Ag30_10s_1240C_1.02kx.tif]

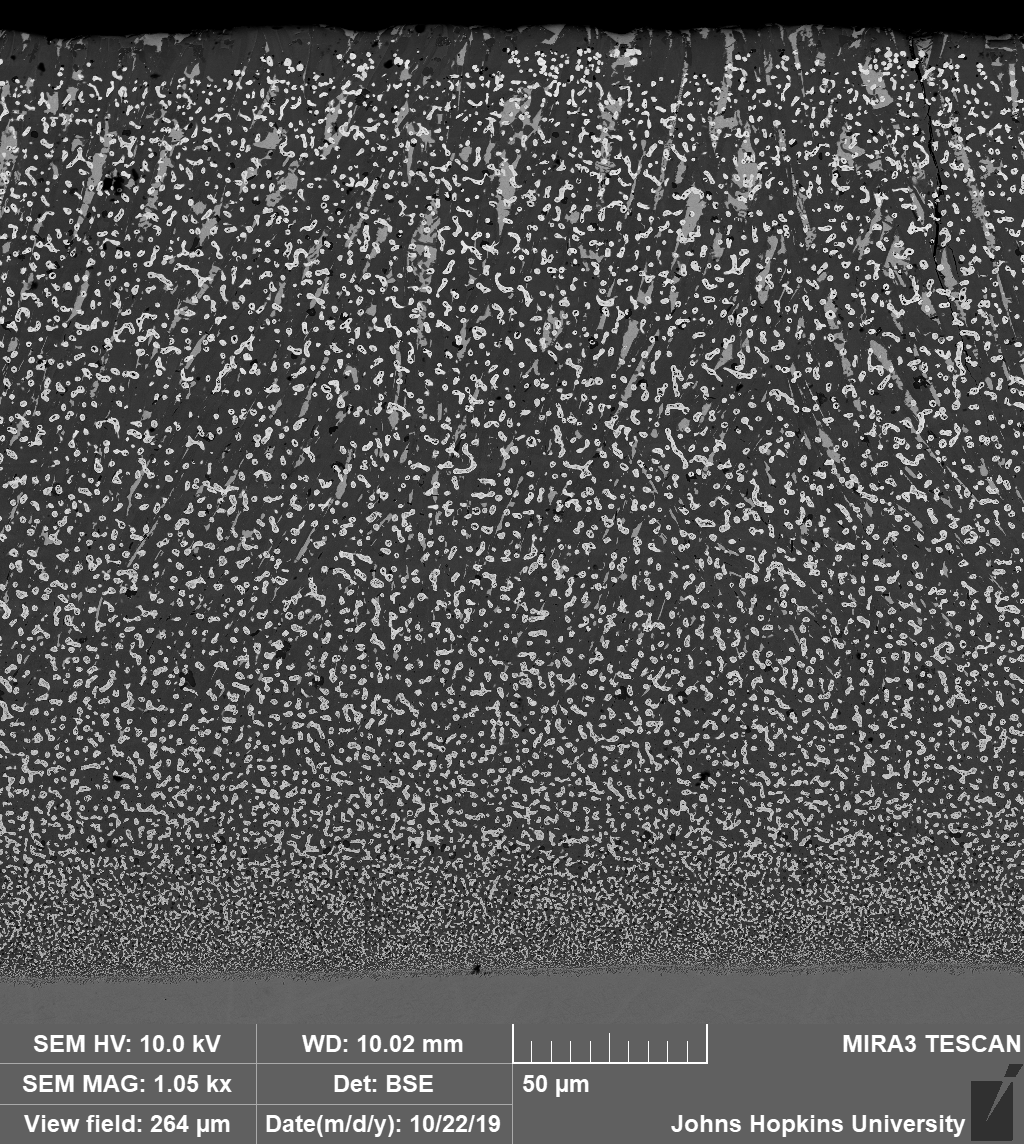

Supplement: Supplementary file 8 — Supplementary Data 1 [file 41467_2022_30483_MOESM8_ESM.zip › 20191022_Ta15Ti85-Cu70Ag30_10s_1240C_1.05kx_EDAX_BSE.tif]
